# Supplementary figures and images for: A whole blood intracellular cytokine assay optimised for field site studies demonstrates polyfunctionality of CD4+ T cells in acute scrub typhus
Source: PLoS Negl Trop Dis. 2023 Mar 24;17(3):e0010905. doi: 10.1371/journal.pntd.0010905 (PMC10075457; doi:10.1371/journal.pntd.0010905)

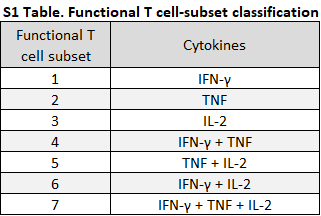

Supplement: S1 Table — (TIF) [file pntd.0010905.s001.tif]

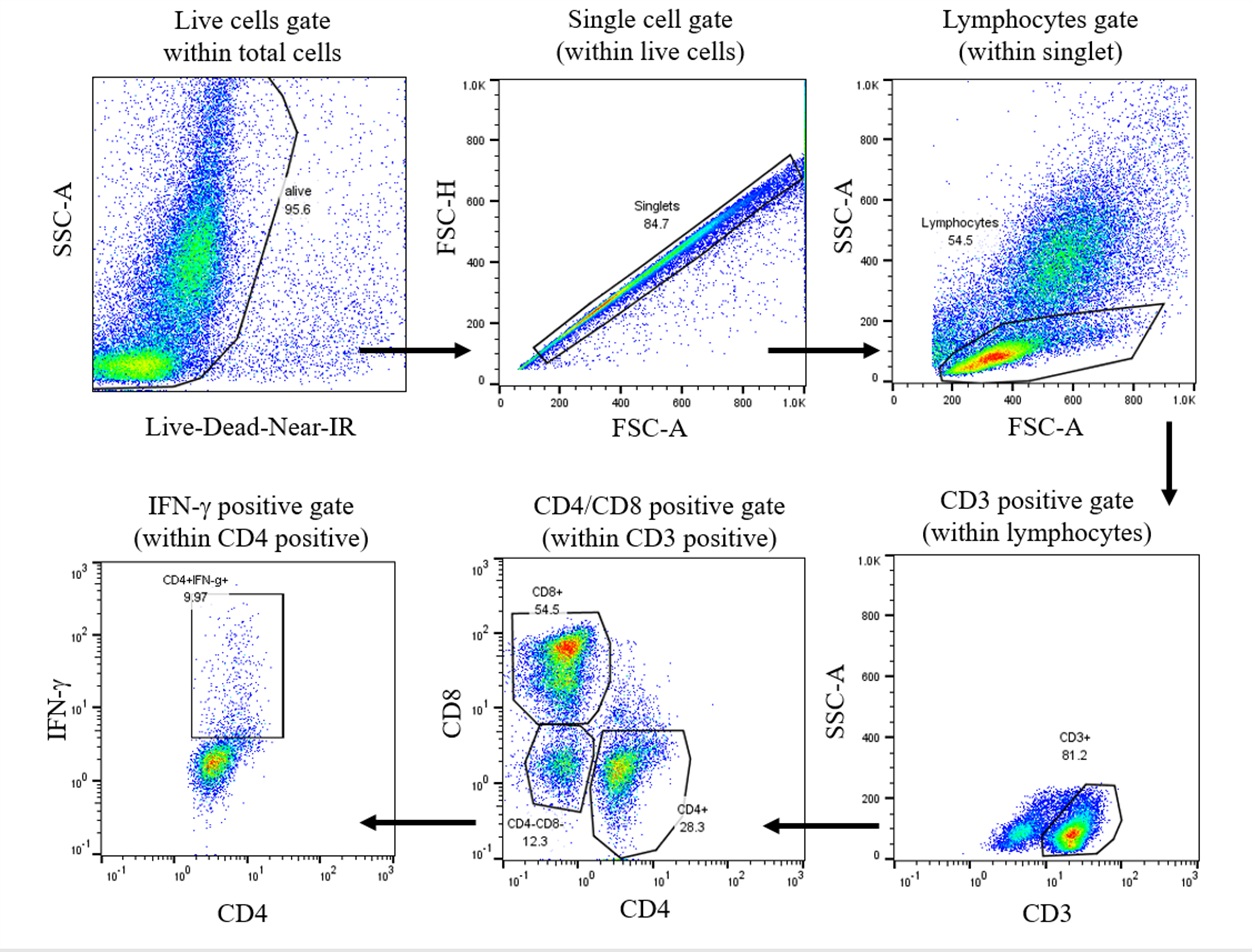

Supplement: S1 Fig — Dead cells were excluded by live-dead cell staining, followed by a single cell gate using forward scatter-area (FSC-A) and height (FSC-H). Lymphocytes were then gated using FSC-A and Side Scatter-area (SSC-A). CD3 positive cells were then selected for further identification of T cell subsets: CD4 or CD8 T positive cells. (TIF) [file pntd.0010905.s002.tif]

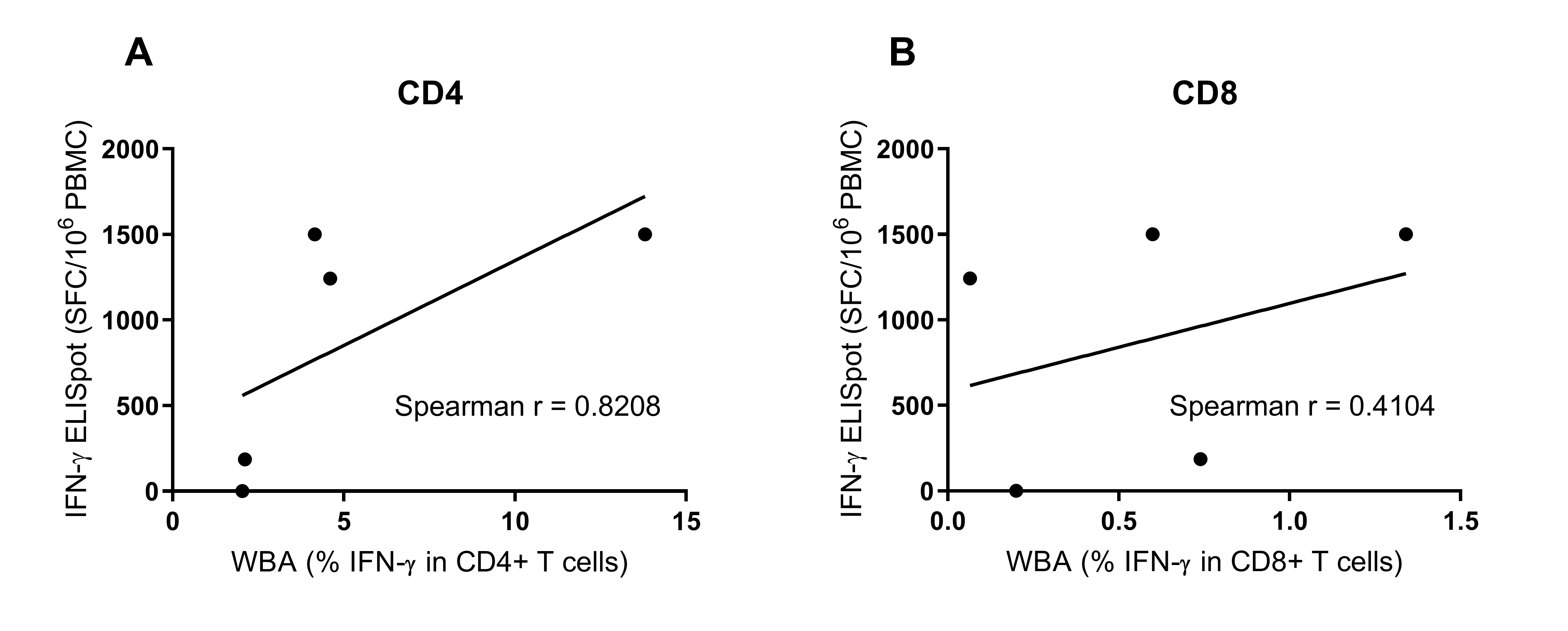

Supplement: S2 Fig — (TIF) [file pntd.0010905.s003.tif]
